# A Review of the Potential Health Benefits of *Nigella sativa* on Obesity and Its Associated Complications

**DOI:** 10.3390/plants12183210

**Published:** 2023-09-08

**Authors:** Siti Hajar Adam, Izuddin Fahmy Abu, Datu Agasi Mohd Kamal, Ami Febriza, Mohd Izhar Ariff Mohd Kashim, Mohd Helmy Mokhtar

**Affiliations:** 1 Preclinical Department, Faculty of Medicine & Defence Health, Universiti Pertahanan Nasional Malaysia, Kuala Lumpur 57000, Malaysia; 2 Institute of Medical Science Technology, Universiti Kuala Lumpur, Jalan Sultan Ismail, Kuala Lumpur 50250, Malaysia; 3 Department of Biomedical Sciences, Faculty of Medicine and Health Sciences, Universiti Malaysia Sabah, Kota Kinabalu 88400, Malaysia; 4 Department of Physiology, Faculty of Medicine and Health Sciences, Universitas Muhammadiyah Makassar, Kota Makassar 90221, Indonesia; 5 Centre of Shariah, Faculty of Islamic Studies, Universiti Kebangsaan Malaysia, Bangi 43600, Malaysia; 6 Insitute of Islam Hadhari, Universiti Kebangsaan Malaysia, Bangi 43600, Malaysia; 7 Department of Physiology, Faculty of Medicine, Universiti Kebangsaan Malaysia, Kuala Lumpur 56000, Malaysia

**Keywords:** *Nigella sativa*, thymoquinone, antioxidants, anti-obesity, body weight

## Abstract

Obesity has become a worldwide epidemic and its prevalence continues to increase at an alarming rate. It is considered a major risk factor for the development of several comorbidities, including type 2 diabetes, stroke, other cardiovascular diseases and even cancer. Conventional treatments for obesity, such as dietary interventions, exercise and pharmacotherapy, have proven to have limited effectiveness and are often associated with undesirable side effects. Therefore, there is a growing interest in exploring alternative therapeutic approaches. *Nigella sativa* (NS), a medicinal plant with multiple pharmacological properties, has gained attention due to its potential role in the treatment of obesity and its associated complications. The aim of this review is therefore to assess the effects of NS on obesity and its complications and to provide insights into the underlying mechanisms. From this review, NS appears to play a complementary or supportive role in the treatment of obesity and its complications. However, future studies are needed to verify the efficacy of NS in the treatment of obesity and its complications and to prove its safety so that it can be introduced in patients with obesity.

## 1. Introduction

In recent years, obesity rates have risen alarmingly worldwide, in both developed and developing countries [1]. It currently affects more than one-third of the world’s population, including both overweight and obese people [2]. If the present patterns persist, it is projected that by 2030 approximately 38% of global adult population will be overweight and another 20% will fall into the obese category [3]. If not properly addressed, the problem could lead to many dreaded complications that increase mortality and morbidity in the population. Obesity is considered an important risk factor for the development of several comorbidities, including type 2 diabetes, stroke, other cardiovascular diseases and even cancer [4,5]. Prevention and management of this problem are crucial to minimise the impact on the global community.

Current treatment strategies for obesity focus primarily on lifestyle changes, including dietary interventions, physical activity, behavioural therapy and pharmacotherapeutic interventions [6]. However, it has been proven difficult to maintain long-term weight loss, as only a small percentage of people (5–10%) are able to sustain their weight loss over time [7,8]. Adopting a plant-based diet that targets lower calorie density has also shown encouraging results in relation to weight loss, leading to lower cholesterol levels and improved insulin sensitivity [9,10]. However, for some people this approach is challenging and cannot be sustained over a long period of time. In addition, there are a variety of pharmaceutical interventions to combat obesity, most of which focus on appetite suppression mechanisms to increase energy expenditure and limit calorie intake [11,12,13]. However, anti-obesity drugs are often associated with various adverse effects and their efficacy decreases after prolonged use [14]. The multifactorial aetiology of obesity requires multiple intervention strategies beyond traditional drug therapies that are better accepted by patients [15]. Suboptimal outcomes have also been observed after discontinuation of lifestyle modifications or pharmacotherapy, highlighting the need for alternative or complementary therapeutic options to achieve better and more durable weight loss outcomes in obesity [16].

Among the modalities of complementary and alternative medicine (CAM), herbal supplements and nutritional therapies have emerged as prominent modalities related to obesity interventions [17]. Extensive research on natural products and medicinal plants, including crude extracts and isolated compounds, has shown that they have the potential to induce weight loss and prevent obesity [18,19,20]. Their efficacy can be attributed to the different phytochemical components they contain, each of which has different effects against obesity and as antioxidants by modulating body metabolism and fat oxidation [21,22]. In addition, medicinal herbs are more accessible, less expensive and generally have fewer side effects than synthetic drugs. One such herb that has shown promise in weight loss and combating obesity is *Nigella sativa* (NS) [23].

NS commonly known as black cumin, is a medicinal plant belonging to the Ranunculaceae family [24]. It is mainly cultivated in the countries of the Middle East and Southwest Asia and has been used in traditional medicine for centuries due to its diverse medicinal properties. NS has been used to treat various diseases for 2000 years [25]. In traditional Arab and Indian culture, it is often used as food and medicine [26,27,28]. In Islamic culture it is called “El Habba Saouda (Seeds of Blessing)” and is known as a medicinal plant traditionally used for all diseases except death [29]. Avicenna (Ibn Sina), a famous 10th century physician and the father of early modern medicine, described several benefits of NS in increasing body energy [23]. 

Numerous studies have NS potential therapeutic effects in diseases such as diabetes mellitus [30,31], cancer [32,33] and neuroprotective activity [34]; and gastroprotective activity [35], cardioprotective activity [36,37], anti-dyslipidaemia and anti-obesity activity [38], to name a few. In addition, NS has anti-inflammatory activity, inhibits proinflammatory cytokines and eosinophils in rheumatoid arthritis [39] and responds well to the treatment of psoriasis [40]. In terms of antioxidant activity, the fatty acid compound in NS is able to suppress the excessive formation of ROS [41]. NS has also responded well to anti-cancer activity, as all agents have a positive anti-cancer effect [33]. It has induced apoptosis in blood cancers [42] and it has significantly reduced the viability of breast cancer cells [43]. There are several bioactive compounds present in NS, including thymoquinone (TQ), thymohydroquinone and dithymoquinone, which have anti-inflammatory and antioxidant properties and contribute to NS various pharmacological potentials [24]. These compounds target multiple signalling pathways involved in adipogenesis [44], lipid metabolism [45] and appetite regulation [46], making NS a potential alternative intervention for obesity.

While several reviews have examined the broad pharmacological effects of NS, there is little literature that specifically addresses its effects on obesity and its associated complications. To address this research gap, the aim of this study is to comprehensively review the existing literature on the potential therapeutic benefits of NS on obesity and its associated complications. This includes a comprehensive analysis of data derived from various animal models, clinical trials and in vitro studies. This review will contribute to the existing body of knowledge by consolidating the available evidence and providing a basis for future studies aimed at harnessing the therapeutic potential of NS in the fight against obesity. Understanding the potential benefits and limitations of NS as a complementary or alternative treatment for obesity could pave the way for the development of new therapeutic strategies.

## 2. Methods

A literature search was conducted to identify and present relevant articles related to the effects of NS on obesity. Peer-reviewed full-text English articles up to May 2023 were collected from electronic databases, including Scopus, MEDLINE via EBSCOhost and Google Scholar. The following keywords were used: (1) “*Nigella sativa*” or “*Nigella sativa* oil” or “*Nigella sativa* extracts” and (2) “obesity”. The literature search was supplemented with references to related review articles and scientific reports found in the search results. All studies, including in vitro, in vivo and human studies, reporting the effects of NS or NS oil or NS seeds on parameters related to obesity and its associated complications were included in this review. On the other hand, studies on NS, which did not address parameters of obesity and related complications were excluded from this study. 

## 3. Results and Discussions

### 3.1. Chemical Composition of Nigella sativa

Numerous chemical compounds have been identified in NS. The compounds may vary depending on the area of cultivation, degree of ripeness, processing methods and isolation techniques. Different extraction methods or solvents, such as in oil samples extracted with a cold press, hexane, tetrahydrofuran, ethanol, dichloromethane and methanol, yielded different amounts of chemical compounds as the methods used affected the oil quality [47]. NS consisted of significant amounts of iron, copper, zinc, phosphorus, calcium, thiamine, niacin, pyridoxine, and folic acid [27,48,49]. In addition, it had maximum nutritional value with a significant amount of 20–85% vegetable protein, 7–94% fibre, 38.02% fat and 31.94% carbohydrate [50]. Apart from these, NS also contains bioactive phytochemicals such as terpenes and terpenoids, phytosterols, alkaloids, tocols and polyphenols [23]. Table 1 lists the chemical composition of NS. 

The main constituent of NS, which is considered to have to medicinal value, is TQ [24]. TQ (2-isopropyl-5-methylbenzo-1, 4-quinone) is the main constituent of the volatile oil of the NS seeds [51]. TQ can be found in tautomeric forms, such as the enol form, the keto form, and in combination. The pharmacological properties of this compound are due to the keto form, which constitutes the major part (about 90%) of the compound [51]. TQ is a promising compound for modern pharmacology because it has been shown to act as a modulator in several pharmacological pathways involving inflammatory responses, oxidative markers, apoptosis, peroxisome proliferator-activated receptors (PPARs) and transcription factors [52].

The bioactive compounds in NS, especially TQ and polyphenols such as gallic acid, p-coumaric acid, naringenin and quercetin, are thought to play a role in the inhibitory effect of NS on the digestive enzyme α-amylase and glucose uptake in the intestine, which could explain its properties as an anti-obesity agent [53,54]. Further studies could focus on the chemical compounds in NS that mediate the anti-obesity effect, with a view to possibly extracting them and using them as an anti-obesity agent.

**Table 1:** Lists of the chemical composition of NS.

Component	Composition	References
Fatty acid	Linoleic acid, oleic acid, lauric acid, stearic acid, linolenic acid	[55,56]
Vitamin	Ascorbic acid, tiamin, riboflavin, pyridoxine, niacin	[56,57]
Mineral	Calcium, magnesium, potassium, phosphorus and iron	[58]
Alkaloids	Nigellidine, nigeglanine, nigelanoid, 17-O-(β-d-glucopyranosyl)-4-O-methylnigellidine, 4-O-methylnigeglanine	[59]
Terpenes and Terpenoids	Thymoquinone, thymohydroquinone, dithymoquinone, p-cymene, sesquiterpene longifolene	[23,55]
Polyphenols	Apigenin, naringenin, gallic acid, rutin, quercetin, kaempferol	[60]
Phytosterols	Campesterol, stigmasterol, β-sitosterol	[61]
Tocols	β-tocotrienol, γ-tocopherol isomer, β-sitosterol	[55]
Saponin	Alpha-hederin (α-HN), 3-O-(β-d-xylopyranosyl-(1-3)-α-l-rhamnopyrnaosyl-(1-2)-α-l-arabinopyranosyl]-28-O-(α-l-rhamno-pyranosyl-(1-4)-β-d-glucopyranosyl-(1-6)-β-d-glucopyranosyl] hederagenin, 3-O-[α-L-rhamnopyranosyl-(1-2)-α-L-arabinopyranosyl]-28-O-[α-L-rhamnopyranosyl-(1-4)-β-D-glucopyranosyl-(1-6)-\β-D-glucopyranosyl]-hederagenin and 3-O-[β-D-xylopyranosyl-(1-3)-α-L-rhamnopyranosyl-(1-2)-α-L-arabinopyranosyl]-hederagenin	[62,63,64]

### 3.2. Effects of NS on Obesity and Its Associated Complications

#### 3.2.1. Preclinical Studies

The prevalence of obesity is rapidly increasing worldwide. It is generally accepted that natural products that have been shown to be safe can be used to combat obesity [65]. A previous in vivo study showed that rats treated with NS oils had a significant decrease in body weight (BW) and a lower risk of developing hyperglycaemia and hyperlipidaemia (serum total cholesterol and triglyceride (TG) levels compared to high-fat-diet (HFD) rats [66]. Rats receiving petroleum ether extract from NS for four weeks also showed a 25% decrease in food intake, resulting in transient weight loss. Remarkably, the animals fed NS had lower fasting plasma levels of insulin and TG and higher HDL cholesterol compared to control rats [67]. Another in vivo study demonstrated the therapeutic and protective effects of various extracts of NS (TQ, hydroalcoholic and hexane extracts) against oxidative stress induced by a HFD. The hydroalcoholic and hexane extracts were found to induce weight loss by having a positive effect on UCP-1, the index protein of brown adipose tissue, at gene and protein levels [68]. 

Treatment with NS also reduced BW and food intake without affecting water intake. There is evidence that NS may reduce BW through the mechanism of appetite suppression. The suppression of appetite might be associated with the neuronal circuits responsible for regulating the catecholaminergic, serotonergic and peptidergic systems [69]. Furthermore, the effect could be mediated via the signalling of the hormone leptin in the satiety centre of the brain, leading to hypophagic effects and subsequent weight loss in experimental animals [70]. Previous research has also reported that consuming NS could lead to an increase in ghrelin, one of the peptides that regulate appetite [71]. 

At the same time, HFD intake leads to obesity by promoting positive energy balance and the deposition of visceral fat [20]. In in vivo studies, 6-week administration of NS to obese male albino rats fed HFD resulted in a significant improvement in lipid profile to normal levels and a reduction in final body size, serum atherogenic index (AI) and levels of ALT. Histopathological analysis revealed that treatment with NS significantly improved liver lesions in the livers of HFD rats that previously exhibited steatosis features [72]. It was postulated that oxidative damage to hepatocellular proteins and hepatocellular necrosis leading to abnormal hepatocytes is responsible for the altered liver architecture [73]. The beneficial effects of NS in this study may be due to TQ, the main active ingredient of NS. In addition, a previous study has shown that NS fixed oil has low toxicity with a high LD50 and does not cause histopathological changes in the heart, liver, kidney and pancreas tissues of treated rats [74]. In another study, administration of TQ, the major bioactive quinone produced by NS, in combination with omega-3-ω3 fish oil was shown to reduce obesity-associated insulin resistance and the chronic inflammatory state of obesity in mice with HFD [75].

In another study, obese rats that received an extract of NS black cumin for six weeks had significantly lower blood glucose, serum cholesterol, TG and low-density lipoprotein (LDL) levels and significantly higher levels of high-density lipoprotein (HDL) compared to controls [69]. It is plausible that NS may have contributed to lower blood glucose levels via two potential mechanisms: restoration of glucose homeostasis and improvement of insulin sensitivity of liver cells [67]. 

Obesity is often associated with the build-up of adipose tissue, which is widely recognised as a common phenomenon [76,77,78]. Complex diseases such as obesity and insulin resistance are due to alterations in the production of adipokines by adipose tissue [79]. In a recent study, treatment with NS seed extract at various doses of 100–400 mg/kg showed dependent amelioration of high-fat-diet (HFD)-induced obesity in mice compared to metformin (250 mg/kg) [80]. Fat formation and adipocyte hypertrophy were significantly and dose-dependently reduced by all three doses of the extracts from the seeds of NS. In particular, administration of the extract from the seeds of NS at a dose of 200 mg/kg showed significant inhibition of fat formation and adipocyte hypertrophy induced by a high-fat diet compared to the metformin (250 mg/kg) treated group. These results thus suggest that oral administration of NS seed extracts (400, 200 and 100 mg/kg) shows a dose-dependent trend in improving obesity compared to metformin (250 mg/kg).

An in vitro study has shown that the lipase inhibitor cocktail RAYstat4ns, isolated and purified from the seeds of NS, exerts good lipase inhibitory activity on pancreatic lipase with mixed types of inhibition as well as inhibition of hormone-sensitive lipase. These NS-based lipase inhibitors have the potential to treat obesity and prevent type 2 diabetes by controlling lipolysis and insulin resistance. They also suppress the uptake of dietary fats into the living system [81]. Table 2 shows the summary of preclinical studies of NS against obesity and its complications.

#### 3.2.2. Clinical Studies

BW and waist circumference are the ideal anthropometric predictors of abdominal obesity that can be used to predict cardiovascular disease, type 2 diabetes mellitus, metabolic syndrome and other chronic diseases. In a clinical trial of obese women who followed a low-calorie diet and received 3 g/day NS oil, BW, TG and very low-density lipoprotein (VLDL) levels and waist circumference (WC) decreased significantly at the end of the 8-week study period compared to the placebo group [82]. It is possible that NS produces these effects due to its high unsaturated fatty acid content, antioxidant and anti-inflammatory components. Previously, it was shown that the hypotriglyceridaemic effect of NS could be due to the presence of unsaturated fatty acids [83]. Unsaturated fatty acids may modulate the levels of TG and VLDL by affecting the synthesis and degradation of TG-rich lipoproteins. Moreover, NS antioxidants such as TQ and ter-butylhydroquinone (TBHQ) could prevent lipid peroxidation and improve enzyme function in lipid metabolism [84,85]. Meanwhile, a follow-up study showed that the intake of NS oil (3 g/day) in obese women led to a reduction in body fat mass (BFM) and insulin levels, while adiponectin levels increased compared to the placebo group. Adiponectin is an adipokine that is mainly secreted by adipose tissue and has anti-inflammatory and insulin-sensitising properties. However, no significant changes were observed in body mass index (BMI), insulin sensitivity and nuclear receptor PPAR-γ [86]. Liver enzymes also did not change significantly after the intervention, and none of the participants experienced severe side effects, demonstrating the safety of NS. The findings of this study show that taking NS oil in combination with a low-calorie diet can influence hormone secretion and body composition in obese women. 

As shown in animal studies, the potential mechanisms of NS against obesity could include the reduction in appetite and food intake, as well as the active components of NS, such as TQ and thymol, which can influence fat metabolism and insulin secretion [87]. In addition, phytochemicals have been shown to have the ability to manifest their anti-obesity properties through many pathways, including inhibiting the activity of digestive enzymes such as pancreatic lipase and amylase, thereby modulating appetite and reducing white adipose tissue production [88].

Previous research has shown that central obesity is a risk factor that increases the likelihood of cardiovascular events and is associated with metabolic syndrome, insulin resistance and other metabolic disorders. According to a study of centrally obese men, a daily intake of 3000 mg NS resulted in significant reductions in BW, WC and systolic BP. Although not statistically different from the control group, several biochemical measures, including serum free testosterone, diastolic BP, fasting blood glucose (FBG), TG and HDL cholesterol, uric acid and hs-CRP were reduced [89]. To achieve better results, it has been suggested that the dose should be increased and the duration of the treatment should be prolonged. Meanwhile, in a study on obesity, diabetes and dyslipidaemia, patients treated with 6 weeks of standard treatment (atorvastatin 10 mg/day, metformin 500 mg/twice daily in tablet form) were compared with an experimental group receiving the same treatment with NS oil 2.5 mL/twice daily for the same period. In this study, the experimental group showed significantly better results in total cholesterol, low-density lipoproteins (LDL) and FBG levels. In addition, the NS group also showed a decrease in BW, BMI and abdominal circumference, although the difference was not significant [83]. In a comparative study between obese prediabetics taking NS oil soft gelatine capsules 450 mg twice daily and those receiving metformin 500 mg tablets twice daily, anthropometric (BW, BMI), glycaemic, lipid and inflammatory markers were assessed before and six months after the interventions. It was found that both groups showed equivalent improvements in anthropometric (BW, BMI) and glycaemic indicators. In particular, NS improved the lipid panel and showed a significant reduction in TNF-α levels, in contrast to the metformin group [90]. To determine the exact effect of NS on obesity, further studies should be conducted with more participants, longer treatment periods and different NS doses in both sexes [46]. 

A recent study showed that anthropometric and body composition indices, including BW, BMI, WC, body fat percentage (BFP), BFM and visceral fat area, as well as appetite and satiety were improved in obese and overweight women through the daily consumption of 2000 mg of NS oil for two periods of eight weeks [46]. The anti-obesity effects of NS have been shown to be due to its bioactive constituents, including TQ, thymol, the lipase enzyme, and various unsaturated fatty acids, such as linolenic acid, linoleic acid, oleic acid, arachidonic acid, and eicosadienoic acid [67,91]. Meanwhile, numerous studies have shown that supplementation of NS can improve insulin sensitivity and metabolic status, leading to an improvement in metabolic profile and a significant reduction in BW [66,92,93]. Thus, the findings may suggest that the consumption of NS is a safe adjuvant therapy for the treatment of obesity in adult women. 

In obese people, the level of reactive oxygen species (ROS) is increased and at the same time antioxidant defence mechanisms are reduced. Obesity is associated with increased oxidative stress, which can lead to the initiation and progression of inflammatory processes [94]. It should also be noted that the hormone leptin, which is produced by fat cells, also contributes to the triggering of oxidative stress [95]. Weight loss has been shown to strengthen antioxidant defences [96,97]. A study by Namazi et al. (2015) showed that an eight-week low-calorie diet supplemented with 3 g/day NS oil resulted in a significant reduction in BW and an increase in antioxidant superoxidase dismutase (SOD) in red blood cells in obese women compared to the placebo group [98]. This could be due to the fact that NS has a protective effect against free radical attacks [98]. A previous study showed that treatment of rats with hepatotoxicity-induced lipid peroxidation with 10 mg/kg TQ orally for 5 weeks decreased lipid peroxidation and increased SOD activity in liver tissue [99]. There is also evidence that the synergistic action of the antioxidant components in NS may protect tissues from lipid peroxidation and oxidative stress. The regulation and expression of antioxidant enzyme genes to defend against free radical attack has been proposed as another possible mechanism for the antioxidant effects of NS. In rats suffering from lipid peroxidation caused by hepatotoxicity, Wahab et al. found that TQ (10 mg/kg orally for 5 weeks) increased SOD activity in liver tissue and decreased lipid peroxidation. It has been postulated that TQ has the ability to maintain cell membrane integrity and attenuate the enhanced process of lipid peroxidation [99].

**Figure 1:**
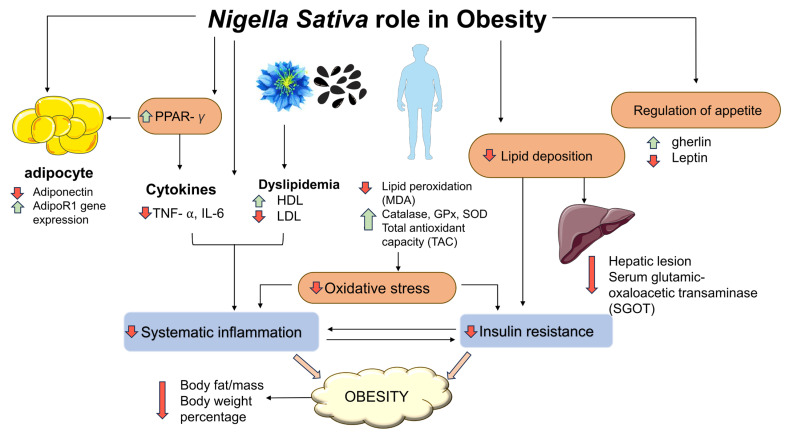
Effects of *N. sativa* in obesity. Solid black lines indicate a mechanism associated with development of obesity, including oxidative stress, systemic inflammation and insulin resistance. The green arrows indicate increased effects and red arrows indicate decreased effects following NS administration. Malondialdehyde (MDA); superoxide dismutase (SOD); glutathione peroxidase (GPx; interleukin (IL); tumour necrosis factor-alpha (TNF-⍺); high density lipoprotein (HDL); low density lipoprotein (LDL).

Another study found that consuming NS supplements helps obese people prevent cardiovascular diseases (CVD). Healthy overweight and obese women showed an overall improvement in their cardiovascular disease risk variables after consuming 2000 mg/day of NS for 8 weeks. Specifically, supplementation of NS also lowered the following parameters: systolic blood pressure (BP), TC/HDL-C ratio, serum glutamic-oxaloacetic transaminase, low-density lipoprotein cholesterol and elevated serum high-density lipoprotein cholesterol [100]. The overall improvement in cardiovascular disease risk factors showed that NS supplements may help prevent possible cardiovascular disease in adults with obesity. A more recent study with a similar subject group and intervention demonstrated that supplementation with NS oil had a positive effect on BW and improved important parameters of adipogenesis and obesity, such as the reduction in transcription levels and blood levels of TNF-*α*; the significant increase in AdipoR1 expression, serum adiponectin, gene expression and serum levels of PPAR-*γ* [44]; the increase in serum total antioxidant capacity (TAC) and the significant decrease in serum malondialdehyde (MDA) levels [101]. Table 3 shows a summary of clinical studies of NS against obesity and its complications. Meanwhile, Figure 1 summarises the effects of NS against obesity and its complications in animal and clinical studies.

### 3.3. Strength and Limitations of Study

The strength of the current review lies in its comprehensive approach, including both experimental and clinical studies, to establish NS as a potential intervention in the treatment of obesity. Nevertheless, it should be remembered that the omission of studies on metabolic syndrome and, to some extent, type 2 diabetes mellitus, which are often associated with obesity, may be a limitation in this review. However, as this review was intended to focus on the experimental model and the topic of obesity, the aforementioned limitation can be considered minor in the context of the defined scope and objectives of the review.

### 3.4. Future Perspective

To verify the effectiveness of NS in the treatment of obesity and its complications, future studies with a larger sample are needed to provide more accurate results and include both sexes. This will provide proof of its safety so that it can be introduced to patients with obesity. Further studies are needed to establish the mechanism of action of NS and its phytochemical compounds. This includes identifying the composition of NS sources, dosage, duration of intervention and the best method of administration to achieve optimal therapeutic benefit. Drug–herb interaction is another key factor to ensure safe administration of NS as a therapeutic intervention for the treatment of obesity. The consideration of these parameters will bring us one step closer to the safe introduction of NS as one of the alternative interventions in the treatment of obesity and its complications.

## 4. Conclusions

In summary, several preclinical studies have demonstrated the anti-obesity effects of NS and its compounds. In animal models treated with extracts of NS or its active constituents, a reduction in body weight, adipose tissue mass and serum fat levels was observed. These effects are thought to be mediated through modulation of adipocyte differentiation, lipolysis, fatty acid synthesis and energy expenditure. In addition, NS has been reported to regulate appetite by affecting neuropeptides involved in the signalling of hunger and satiety. Similarly, clinical studies show that NS has the potential to improve obesity by improving anthropometric parameters, restoring dyslipidaemia and reducing inflammatory markers TNF-a and SOD. Therefore, NS appears to play a complementary or supportive role in the treatment of obesity and its complications. 

## Figures and Tables

**Table 2:** Summary of preclinical studies of NS against obesity and its complications.

Type of Study	Treatment Dosage	Duration of Study	Outcomes of the Study	Reference
**In-vivo study**				
High fat diet induced obese rats	4% NS seeds oil	8 weeks	↓ BW, hyperglycaemia and hyperlipidaemia (TLC and TG levels)	[66]
6-week-old mice (C-57) fed with high fat diet	300 mg/kg NS hexane extract	30 days	↓ BW of mice NS extracts displayed protective and therapeutic effects against oxidative stress, caused by HFD.	[68]
C57BL/6J mice were fed with HFD	0.75% TQ and 2% ω3	8 weeks	↓ Insulin resistance associated with obesity and the chronic inflammatory state of obesity	[75]
Obese rats	NS black seed aqueous extract	6 weeks	↓ BW, food consumption, glucose, serum cholesterol, TG and LDL ↑ HDL	[69]
High-fat diet (HFD)-induced obesity in white male albino rats.	300 mg/kg/day *N. sativa* seeds powder	6 weeks	Normalised the lipid panel ↓ BW, serum AI, and serum ALT Improved hepatic lesions of HFD rats which initially showed steatosis characteristics.	[72]
80-week-old female mice with HFD-induced obesity	200 mg/kg/day NS extract	84 days	↓ BW, fat mass accumulation, ↓ blood glucose, ↓ degenerative renal tubules	[80]
**In-vitro study**				
In vitro study	Lipase Inhibitors from NS		RAYstat4ns exhibited good lipase inhibitory activity against pancreatic lipase with mixed type of inhibition, and inhibition of hormone sensitive lipase for potential treatment against obesity.	[81]

**Table 3:** Summary of clinical studies of NS against obesity and its complications.

Type of Study	Treatment Dosage	Duration of Study	Outcomes of the Study	Reference
Double-blind, randomized placebo-controlled clinical trial; 90 obese women.	3 g/day NS oil intervention	8 weeks	↓ BW, WC, TG and VLDL.	[82]
Prospective study, 60 patients with obesity, diabetes and dyslipidaemia.	Atorvastatin 10 mg/day, tablet Metformin 500 mg/twice a day and NS oil 2.5 mL/twice daily	6 weeks	↓ total cholesterol, VLDL and FBG.	[83]
Double-blind, randomized placebo-controlled clinical trial; 50 obese women.	3 g/day NS oil intervention	8 weeks	↓ BFM and insulin levels. ↑ Adiponectin levels. No serious side effects. No altered liver enzyme levels.	[86]
Double blind test with placebo control, pre-test and post-test design; intervention among 39 central obesity men.	3000 mg of NS	3 months	↓ BW, WC, and systolic BP.	[89]
Open-label, randomized, prospective, comparative study; 70 obese prediabetic subjects.	NS oil soft gelatine capsules 450 mg twice daily	6 months	Improved anthropometric and glycaemic parameters and lipid panel. ↓ TNF-α,	[90]
Crossover, double-blind, placebo-controlled RCT; 39 obese and overweight women.	2000 mg/d NS	Two 8-week periods of intervention and a 4-week wash-out period	↓ BMI, BW, WC, BFP, BFM, visceral fat area and feeling of appetite.	[46]
Double-blind, randomized placebo-controlled clinical trial; 49 obese women.	3 g/day NS oil intervention	8 weeks	↓ BW. ↑ SOD levels.	[98]
Crossover, randomised-controlled trial; 39 obese and overweight healthy women.	2000 mg/day of NS oil	8 weeks separated by a 4-week washout period	↑ serum HDL. ↓ LDL, TC/HDL-C ratio, serum glutamic-oxaloacetic transaminase and systolic BP.	[100]
Secondary analysis of a crossover, double-blind, randomized clinical trial; 39 obese and overweight healthy women.	2000 mg/day of NS supplement	8 week intervention separated by a 4-week washout period	↑ serum TAC. ↓ serum MDA.	[101]
A crossover, randomised-controlled trial, overweight/obese women.	2000 mg/d) of NS Oil	Two periods of intervention (8 weeks in each) were cross-changed by a 4-week washout period	↓ BW, transcription levels and blood concentrations of TNF-*α.* ↑ AdipoR1 expression and serum adiponectin and gene expression and serum levels of PPAR-*γ*.	[44]
